# Enhancing Staphyloxanthin Synthesis in Staphylococcus aureus Using Innovative Agar Media Formulations

**DOI:** 10.7759/cureus.59892

**Published:** 2024-05-08

**Authors:** Nirmala B, Balram J Omar

**Affiliations:** 1 Microbiology, All India Institute of Medical Sciences, Rishikesh, Rishikesh, IND

**Keywords:** staphyloxanthin, pigment, media, carotenoid, beetroot, carrot

## Abstract

Background

Staphyloxanthin, a carotenoid pigment found in *Staphylococcus aureus*, serves not only to impart color but also functions as a crucial antioxidant contributing to virulence. Traditionally, milk agar has been employed to enhance staphyloxanthin production, however, no alternative media have been explored.

Objectives

This study aims to enhance staphyloxanthin production in *Staphylococcus aureus *using beetroot and carrot formulations.

Methods

To assess the efficacy of the media, we utilized filter paper, slide spot tests, and microscopic visualization as preliminary identification techniques. Ultraviolet-visible (UV-Vis) spectroscopy and paper chromatography were employed for characterization. Pigment quantification was conducted using microtiter plate assays, and genotypical detection was performed using Reverse Transcriptase-quantitative Polymerase Chain Reaction (RT-qPCR).

Results

Beetroot agar exhibited the highest pigment intensity, followed by beetroot with carrot agar, milk agar, carrot agar, and nutrient agar with the lowest intensity. These novel media formulations increased staphyloxanthin synthesis yield, resulting in spectrum shifts ranging from 450 nm (yellow) of milk agar to 470 nm (carrot agar) /480 nm (orange) of beetroot agar.

Conclusion

This study demonstrates that beetroot and carrot agar can effectively enhance staphyloxanthin production in *Staphylococcus aureus*. Furthermore, we propose the potential for large-scale cultivation of these pigments in future studies for various industrial applications, such as integration into paints, fabrics, and sunscreen lotions, due to their antioxidant properties.

## Introduction

Carotenoids are vibrant pigments naturally occurring in various organisms, including bacteria, archaea, fungi, algae, and plants, impart hues of yellow, orange, and red to a plethora of foods such as pumpkins, corn, carrots, tomatoes, and even avian species like flamingos. Structurally derived from tetraterpenes, these pigments contribute significantly to the visual appeal of these organisms [1]. Numerous bacterial species, such as *Xanthophyllomyces dendrorhous*, *Haloferax alexandrines*, *Staphylococcus aureus*, and others [2], alongside cyanobacteria and fungi like *Phycomyces blakesleeanus* and* Rhodosporidium*, are known producers of carotenoid pigments [3]. Many of these microbial pigments, including riboflavin from *Bacillus subtilis* (E101 iii) and β-carotene from *Blakeslea* (E160a ii), are utilized as food coloring agents [4]. Of particular interest is staphyloxanthin, a golden carotenoid pigment synthesized by *Staphylococcus aureus*, which lends the species its name ('aureus' meaning 'golden' in Latin).

The spectrum of diseases caused by *S. aureus* ranges from mild skin infections to more severe conditions including food poisoning, bacteremia, endocarditis, meningitis, pneumonia, soft-tissue infections, bone and joint infections, medical implant infections, and co-infections [5]. Staphyloxanthin production is regarded as a significant virulence factor of *S. aureus*, alongside various other factors including enzymes such as coagulase, catalase, lipase, protease, DNase, hyaluronidase, beta-lactamase, as well as toxins such as toxic shock syndrome toxin 1 (TSST-1), exfoliative toxin, Panton-Valentine Leucocidin (PVL), alpha, beta, and delta toxin, and the Type VII Secretion System (T7SS). Additionally, *S. aureus* virulence encompasses biofilm formation, immunoinvasive strategies, and antibiotic resistance [5]. Structurally, staphyloxanthin is a β-D-glucopyranosyl 1-O-(4,4′-diaponeurosporen-4-oate)-6-O-(12-methyltetradecanoate). Its synthesis is regulated by the crtOPQMN operon [6].

Staphyloxanthin synthesis begins with the conversion of farnesyl diphosphate into dehydrosqualene catalyzed by the enzyme dehydrosqualene synthase (CrtM). Subsequently, dehydrosqualene undergoes further transformations, with dehydrosqualene desaturase and diaponeurosporene oxidase sequentially converting it into diaponeurosporene and diaponeurosporenic acid, respectively. Diaponeurosporenic acid is then enzymatically modified into diaponeurosporenoate, followed by its subsequent conversion into staphyloxanthin through glycosyl transferase and acyl transferase [6].

Staphyloxanthin serves as an antioxidant, aiding *S. aureus *in combating free radicals such as hydrogen peroxide and hydroxyl radicals [7], thus enhancing its survival in hostile environments, resisting neutrophils, and contributing to the formation of abscesses [8]. Sakai et al. explored inhibitory substances against staphyloxanthin production and determined that lipid inhibitors such as cerulenin, dihydrobisvertinol, xanthohumol, zaragozic acid, as well as two actinomycete metabolites, methylrabelomycin, and tetrangomycin, effectively hindered staphyloxanthin production, thereby aiding in infection reduction [9]. Various culture media were employed to boost pigment production in *S. aureus*, including milk agar, trypticase soy agar (TSA), peanut seed agar, sunflower seed agar, brain-heart infusion agar (BHI), and nutrient agar. Among these, milk agar notably yielded superior pigment enhancement and emerged as the preferred choice for *S. aureus *pigment production [10]. Previous studies investigated the augmentation of staphyloxanthin pigment production in *S. aureus* using gum acacia at a concentration of 1g/dl, owing to its polysaccharide and glycoprotein constituents. However, a higher 2g/dl concentration inhibited pigment formation [11]. As of now, our understanding suggests that there's limited exploration into alternative strategies for increasing staphyloxanthin production. Hence, this study aims to discover improved alternative media for enhancing staphyloxanthin production. In our investigation, we utilized beetroot and carrot agar to stimulate pigment production in *S. aureus*. These media were observed to augment pigment production and yield distinct shades (spectrum ranging from 450 nm to 480 nm) compared to milk agar. Consequently, these findings suggest the potential of utilizing beetroot and carrot agar to amplify staphyloxanthin production in *S. aureus* which can be used for industrial purposes in future studies.

## Materials and methods

Preparation of media

**Figure 1:**
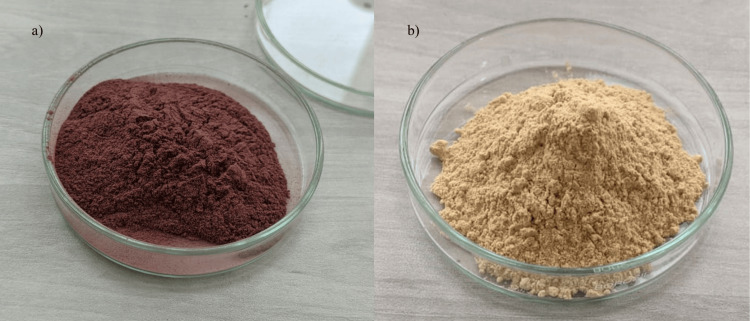
Powder preparation a) Beetroot powder, b) Carrot powder.

**Figure 2:**
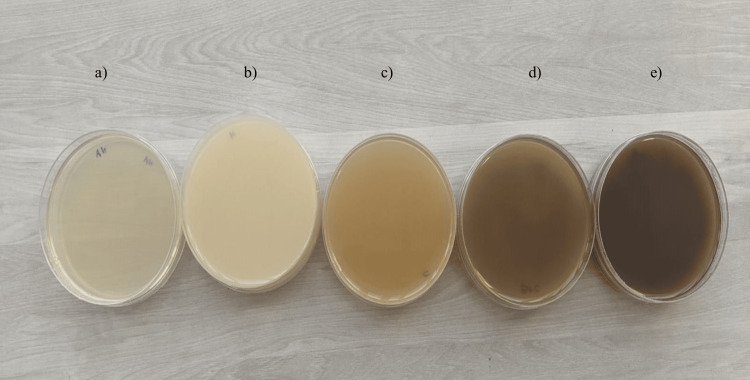
Media preparation a) Nutrient agar, b) Milk agar, c) Carrot agar, d) Beetroot with carrot agar, e) Beetroot agar

To prepare beetroot and carrot powder, whole beetroot and carrot were thinly sliced and subjected to shade-drying in an incubator at 37°C for five to seven days until fully dehydrated. Subsequently, the dried slices were finely ground into powder (refer to Figure 1) and stored in an air-tight container. To prepare beetroot agar, carrot agar, and beetroot with carrot agar, 10 g of beetroot powder, 10 g of carrot powder, and 5 g of beetroot powder with 5 g of carrot powder respectively were added to basal media (5 g peptone, 5 g sodium chloride, 1.5 g HM peptone (HiMedia Laboratories LLC, PA, USA), 1.5 g yeast extract, and 15 g agar to make 1 L ), with final pH of 7.4 ± 0.2. To prepare 100 ml of each agar type, 3.8 g of the corresponding powder was dissolved in 100 ml of distilled water and autoclaved at 121°C for 15 minutes at 15 psi. The resulting media were poured into disposable polystyrene plates and allowed to solidify. Once solidified, the plates were stored at 4°C until further use. Additionally, milk agar and nutrient agar were prepared according to the manufacturer's instructions (HiMedia Laboratories LLC, PA, USA) for comparison purposes (refer to Figure 2).

Bacterial collection, identification, and inoculation of the medium

**Figure 3:**
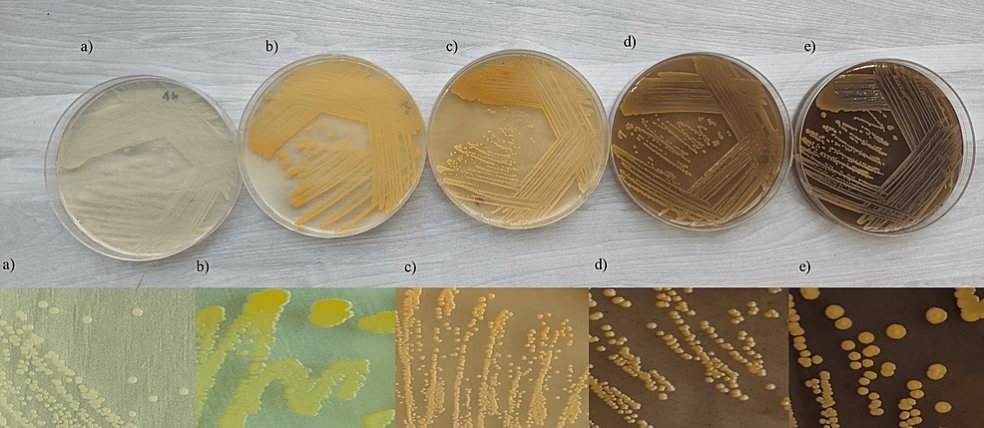
Plates after pigment production S. aureus showing pigment a) Nutrient agar, b) Milk agar, c) Carrot agar, d) Beetroot with carrot agar, e) Beetroot agar

*S. aureus* clinical isolates and the ATCC 25923 strain were sourced from the bacteriology laboratory from August 2023 to December 2023 at All India Institute of Medical Sciences, Rishikesh. Ethical clearance was obtained from the Institutional Ethics Committee under Letter No. AIIMS/IEC/23/294. Identification procedures included standard biochemical tests, the VITEK 2 automated ID system (BioMérieux, USA), and confirmation through Matrix-Assisted Laser Desorption/Ionization-Time of Flight (MALDI-TOF) analysis (utilizing Bruker’s MALDI Biotyper microbial identification system). A loopful of *S. aureus* colony was streaked onto agar plates including carrot agar, beetroot with carrot agar, beetroot agar, nutrient agar, and milk agar. These plates were then incubated at 37°C for 24-48 hours, after which the intensity of pigment production was compared across each plate (refer to Figure 3). All experiments in this study were conducted a minimum of three times to ensure reproducibility.

Analysis of pigment intensity using preliminary tests

An initial assessment of pigment intensity was conducted utilizing the filter paper method, slide spot test, and light microscopy. In the filter paper method, a loopful of colonies was applied onto filter paper from each agar plate. Colony spots were deposited onto slides for the slide spot test, where pigment intensity was assessed visually. Additionally, for the microscopic examination, a loopful of colonies was spread onto glass slides and observed under 10x magnification to evaluate pigment intensity.

Extraction of staphyloxanthin pigment

**Figure 4:**
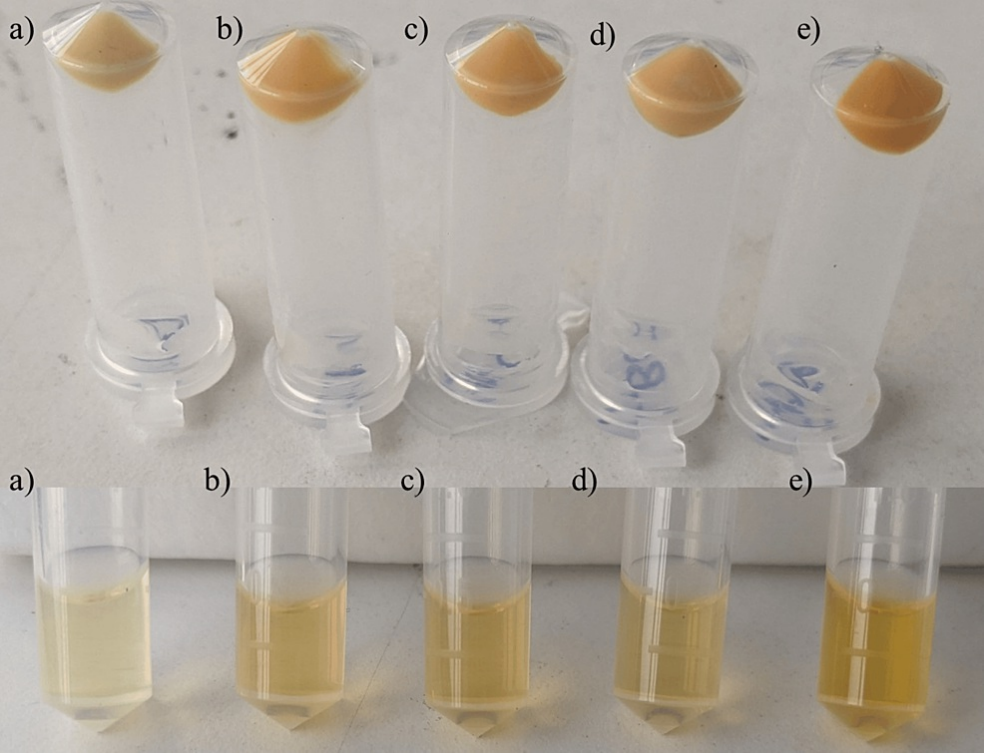
Staphyloxanthin extraction from various media a) Nutrient agar, b) Milk agar, c) Carrot agar, d) Beetroot with carrot agar, e) Beetroot agar. Note the different shades of staphyloxanthin exhibited in different media.

Bacterial cells harvested from each plate were suspended in a saline solution. The optical density (OD_600_) was adjusted to 3 McFarland units using the DensiCHEK Plus instrument (BioMérieux, USA), following which 2 ml of the suspension was subjected to centrifugation at 13,000 revolutions per minute (RPM) for five minutes. The resulting pellet was resuspended in 200 μl of methanol and heated at 55°C for five minutes. After another round of centrifugation at 13,000 RPM for five minutes, the supernatant was collected (refer to Figure 4) for concentration measurement using a microtiter plate reader. This process was repeated thrice, and the final volume was adjusted to 3.5 ml using methanol for UV-visible spectroscopy characterization [12].

UV-visible spectroscopy

The pigment extracts underwent characterization using an Ultraviolet-Visible-Near Infrared (UV-VIS-NIR) Spectrophotometer (Agilent Cary 5000, CA, USA) equipped with an absorption range spanning from 300 to 550 nm [6]. To determine the maximum absorption peak, 3 ml of the sample was loaded into 10 mm quartz cells alongside methanol as a reference, and the procedure was conducted following the manufacturer's protocol provided by Agilent Technologies.

Microtiter plate method

Each agar plate's extracted pigment, measuring 100 µl was introduced into individual wells of a 96-well U-bottomed polystyrene microtiter plate. Subsequently, the absorbance was assessed at 460 nm using a microtiter plate reader (Bio Tek Gen 5; Agilent, CA, USA) [13].

Paper chromatography

The extracted staphyloxanthin pigment was subjected to air drying at 37°C to facilitate the evaporation of methanol, resulting in its conversion into powder form. Subsequently, the pigment was applied onto Whatman filter paper 1 and allowed to dry. For chromatographic analysis, a glass container was filled with methanol, ensuring the solvent level remained below the origin spot by maintaining a depth of less than 1 cm. Following this, the dried filter paper was carefully positioned within the container and covered with a lid. Once the solvent front reached approximately 3/4th of the total distance, the filter paper was removed, and the distances traveled by both the solvent front and the solute were immediately noted. These measurements were utilized to calculate the retardation factor (Rf), which was then compared with standard values [14].

Detection of staphyloxanthin gene expression

RNA Isolation

*S. aureus* colonies were initially inoculated onto blood agar and incubated for 24 hours at 37°C. Subsequently, following 24-hour growth, a loopful of colonies from the blood agar were transferred onto carrot agar, beetroot with carrot agar, beetroot agar, nutrient agar, and milk agar, followed by further incubation for 18 hours at 37°C. Bacterial suspensions were then prepared using normal saline, with the optical density at 600 nm (OD_600_) adjusted to 0.5 McFarland units using the DensiCHEK Plus instrument. From this suspension, 2 ml was extracted and subjected to centrifugation at 13,000 RPM for five minutes at 4°C to yield a bacterial pellet. The obtained pellet was utilized for RNA isolation utilizing the HiPurA Bacterial RNA Purification Kit (HiMedia Laboratories LLC, PA, USA) as per the manufacturer's instructions. Briefly, 100 µl of lysozyme was added to the pellet, followed by incubation at 37°C for 30 minutes with intermittent vortexing to disrupt the bacterial cells. Subsequently, 350 µl of RNA lysis solution containing beta-mercaptoethanol was added, and the mixture was transferred to a Hishredder column, followed by centrifugation at 13,000 RPM for two minutes at 15°C. The eluate was collected, and 280 µl of ethanol was added. After thorough mixing, the suspension was transferred to a spin column and centrifuged at 10,000 RPM for one minute at 15°C. The flow-through was discarded, and the column was washed successively with 700 µl of prewash solution and 500 µl of diluted wash solution, followed by centrifugation at 10,000 RPM for one minute and two minutes, respectively, at 15°C. Finally, 50 µl of elution solution was added to the column, followed by centrifugation at 10,000 RPM for one minute at 15°C. The eluted RNA was stored at -80°C until further analysis.

RNA Concentration and Purity

RNA quantification and purity assessment were conducted using the Tecan NanoQuant Plate (Infinite®200 Pro; Tecan Life Sciences, Switzerland) along with the i-control^TM^ nanoquant software (Tecan Life Sciences). To initiate the process, 2 µl of isolated RNA was dispensed into a well of the NanoQuant plate, with the application mode set to RNA. Subsequently, the absorbance ratio at A260/A280 was determined, where a ratio close to 2.0 indicates high RNA purity. Additionally, the concentration of RNA was measured in nanograms per microliter (ng/µl).

Reverse Transcription (RT)

The reverse transcription process was carried out using the cDNA synthesis kit, adhering to the manufacturer’s instructions provided by GBiosciences (St. Louis, MO, USA). In summary, the RT reaction mixture (10 µl) was prepared, consisting of 2X RT Easy Mix (5 µl), random primer (0.5 µl), oligo (dT) 18 primer (0.5 µl), template RNA (total RNA < 2.5 µg), and RNase-free double distilled water (adjusted to a total volume of 10 µl). The mixture was thoroughly mixed using a benchtop mini centrifuge. For the RT reaction, the Applied Biosystems Veriti 96-Well Thermal Cycler (Thermo Fisher Scientific, Waltham, Massachusetts) was employed, following program step one (Reverse transcription) at 42°C for 20 minutes and step two (Inactivation) at 85°C for 5 minutes. Subsequently, the synthesized cDNA was stored at -20°C until further utilization.

Oligonucleotide Primers

**Figure 5:**
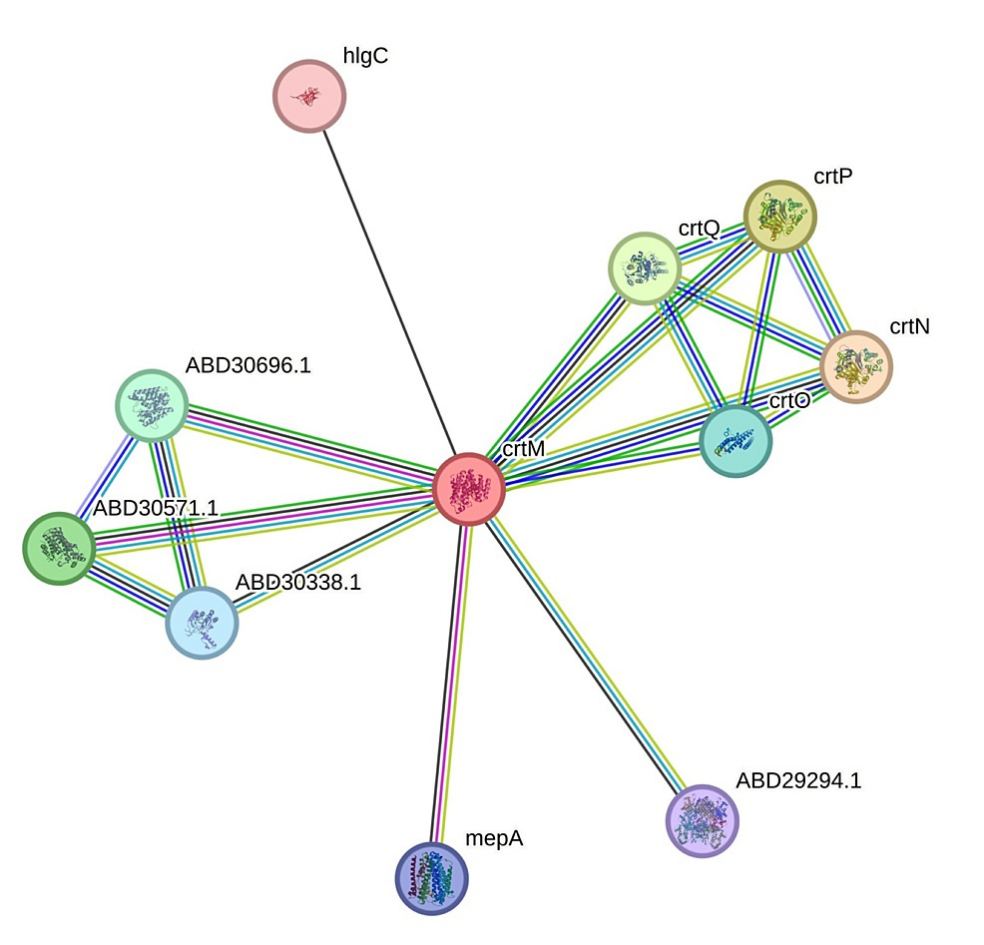
CrtM and its linking protein for staphyloxanthin production (STRING Database) STRING: functional protein association networks (string-db.org)

**Figure 6:**
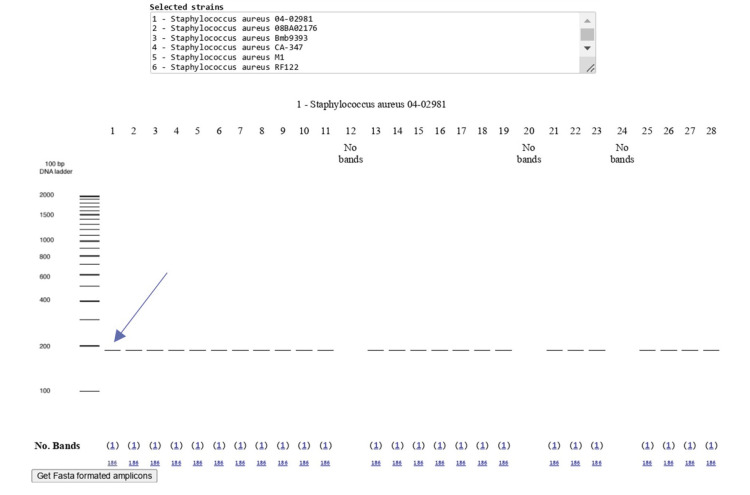
In silico PCR amplification PCR: Polymerase chain reaction.

A primer was specifically designed for the crtM gene, which encodes the dehydrosqualene synthase enzyme involved in staphyloxanthin production (refer to Figure 5), utilizing the NCBI Primer-BLAST tool. The designed primer yielded a product size of 186 base pairs, with the forward primer sequence being 5′-AGAAAAGCGGTTTGGGCAAT-3′ and the reverse primer sequence being 5′-TTGTGCAACATGCTGAAGGG-3′. As an internal control, the *S. aureus* 16S rRNA gene was utilized. After the primer design, the primer sequence was evaluated using an In silico polymerase chain reaction (PCR) amplification computational tool to generate theoretical PCR results (refer to Figure 6).

Quantitative Polymerase Chain Reaction (qPCR) Assay

Quantitative PCR (qPCR) was performed using SYBR^TM^ Green qPCR Master Mix following the manufacturer's instructions provided by G Biosciences. In summary, the qPCR master mix (20 µl) was prepared, consisting of 2X AB HS SYBR Green qPCR Mix (10 µl), forward primer (2 µl), reverse primer (2 µl), cDNA template (2 µl), and double distilled water (4 µl). The mixture was thoroughly mixed using a benchtop mini centrifuge. For the qPCR reaction, the C1000 Touch Thermal Cycler (BIO-RAD CFX96 Real-Time System; Bio-Rad Laboratories, Hercules, CA) was utilized, employing the following thermocycling conditions: initial denaturation at 94°C for two minutes, denaturation at 94°C for 30 seconds, annealing at 54°C for 30 seconds, and extension at 72°C for one minute, repeated for 35 cycles.

Bioinformatics and statistical analysis

The primer sequence was crafted utilizing the National Center for Biotechnology Information (NCBI)'s primer designing tools (https://www.ncbi.nlm.nih.gov/tools/primer-blast/), and its efficacy was assessed via the In silico PCR amplification computational tool (http://insilico.ehu.es/PCR/). The structure of staphyloxanthin was sourced from the Lipid Maps Structural Database (https://lipidmaps.org/), while the structures of betaxanthin and beta-carotene were obtained from the PubChem Structural Database (https://pubchem.ncbi.nlm.nih.gov/). CrtM protein interactions retrieved from the STRING database (https://string-db.org/). UV-Vis spectroscopy graphs were created using Cary WinUV software (Agilent), and absorbance graphs were generated through the Power BI tool. Quantitative PCR graphs were produced using CFX Manager^TM ^software (Bio-Rad Laboratories). Statistical analysis, including correlational coefficient analysis, was conducted using IBM SPSS software, version 21.0 (IBM Corp., Armonk, NY), with statistical significance defined as a p-value < 0.05.

## Results

Preliminary tests

**Figure 7:**
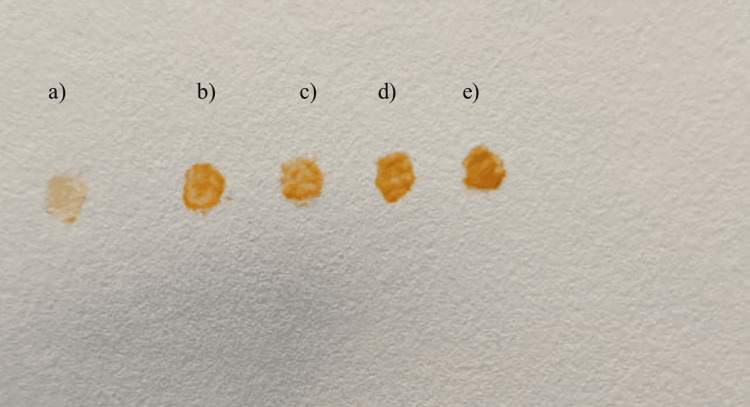
Filter paper test results a) Nutrient agar, b) Milk agar, c) Carrot agar, d) Beetroot with carrot agar, e) Beetroot agar.

**Figure 8:**
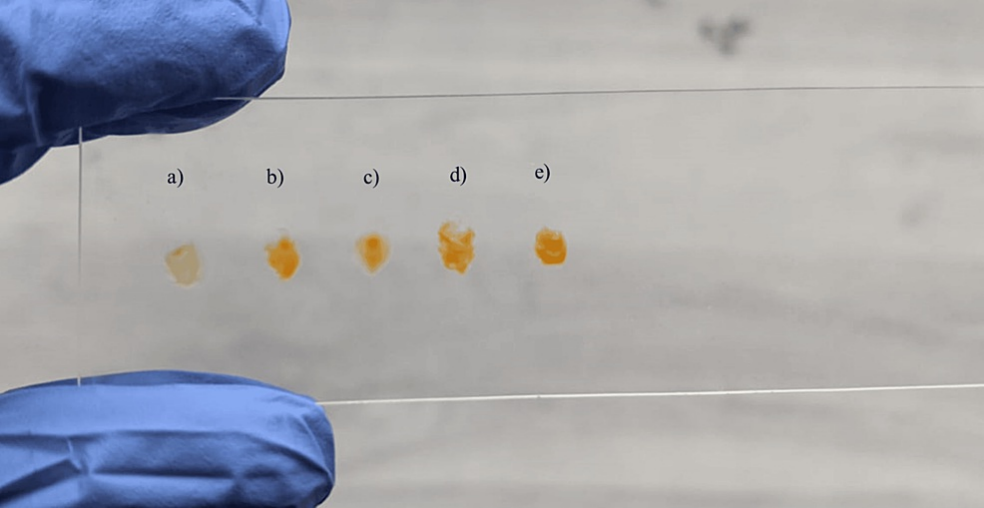
Slide spot test results a) Nutrient agar, b) Milk agar, c) Carrot agar, d) Beetroot with carrot agar, e) Beetroot agar.

**Figure 9:**
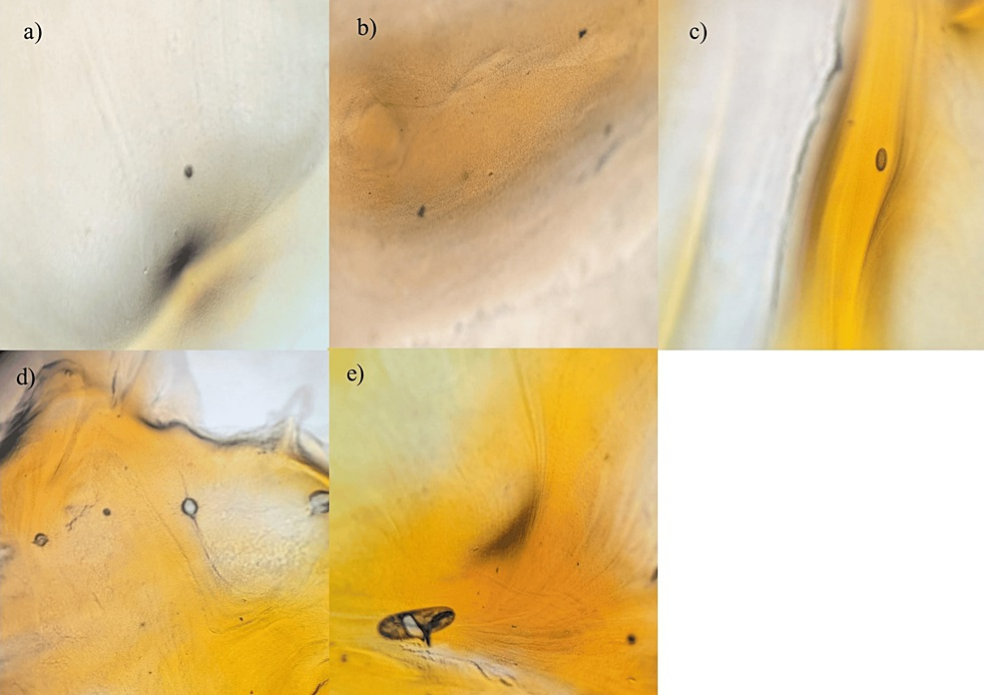
Pigment intensity at 10X magnification a) Nutrient agar, b) Milk agar, c) Carrot agar, d) Beetroot with carrot agar, e) Beetroot agar.

Initial assessments, including the filter paper test, slide spot test, and light microscopy examination, show that beetroot agar exhibits the highest intensity, followed by beetroot with carrot agar, milk agar, carrot agar, and nutrient agar being the least (refer to Figures 7-9).

UV-VIS-NIR spectroscopy characterization

**Figure 10:**
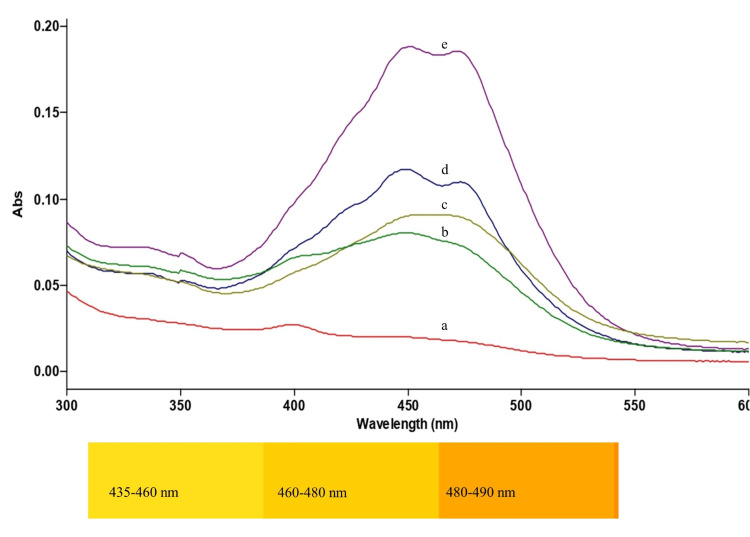
UV-Visible spectroscopy analysis of the extracted pigment a) Nutrient agar at 450 nm (negligible staphyloxanthin concentration), b) Milk agar showing a peak at 450 nm, c) Carrot agar showing a peak at 450 nm - 470 nm, d) Beetroot with carrot agar showing two peaks at 450 and 480 nm, e) Beetroot agar showing two peaks at 450 and 480 nm.

The UV-VIS-NIR spectrophotometer characterization of the pigment extracts reveals distinct patterns: the extracts from nutrient agar display negligible staphyloxanthin peaks at 450 nm. Conversely, milk agar exhibits a prominent peak at 450 nm, indicative of its yellow color, which corresponds to the complementary color perceived by the human eye. Carrot agar displays a peak in the range of 450 nm to 470 nm, signaling a yellow-orange hue, while beetroot with carrot agar and beetroot agar showcase dual peaks near 450 nm and 480 nm, suggesting yellow and orange colors, respectively (refer to Figure 10).

Quantitative analysis of staphyloxanthin

**Figure 11:**
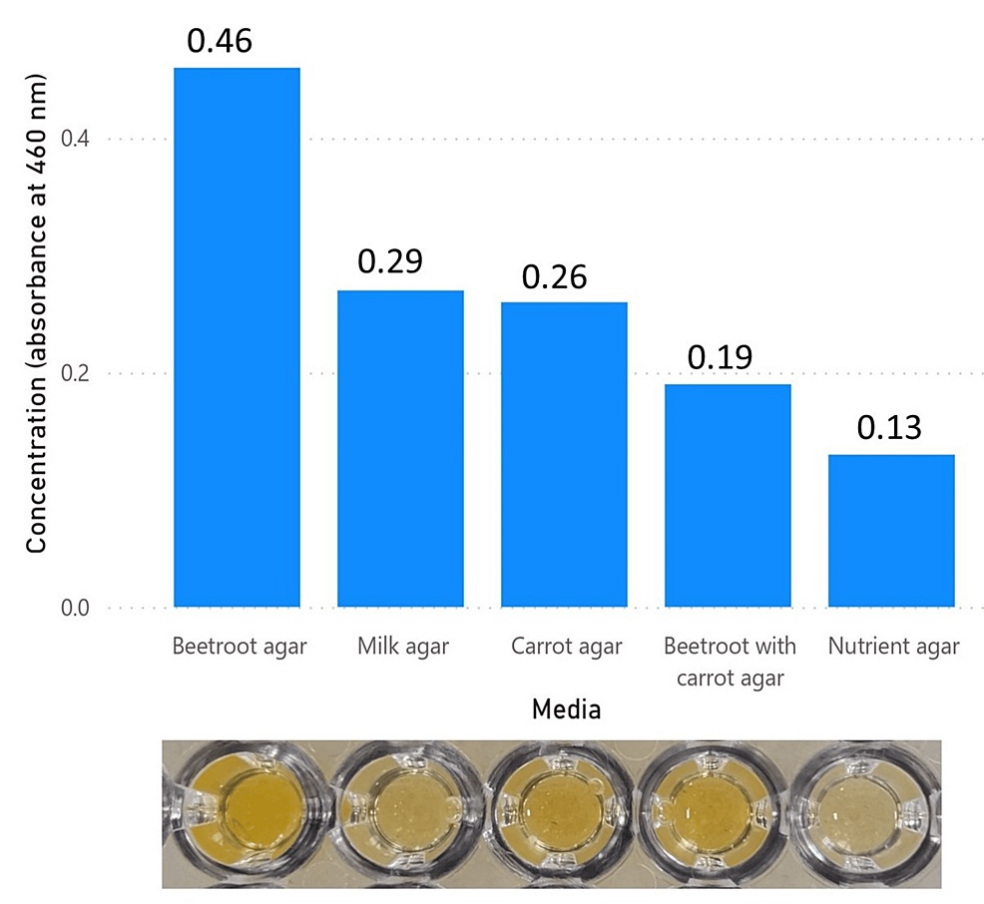
Concentration (absorbance at 460 nm) of the extracted pigment from various media

The various concentrations of staphyloxanthin pigment extracted were measured at absorbance 460 nm using microtiter plate assay revealing nutrient agar (0.13), beetroot with carrot agar (0.19), carrot agar (0.26), milk agar (0.29), beetroot agar (0.46) (refer to Figure 11). Correlation coefficient analysis indicates a strong positive correlation between pigment concentration and incubation time (r = .93, p-value < 0.01).

Characterization of staphyloxanthin by paper chromatography

**Figure 12:**
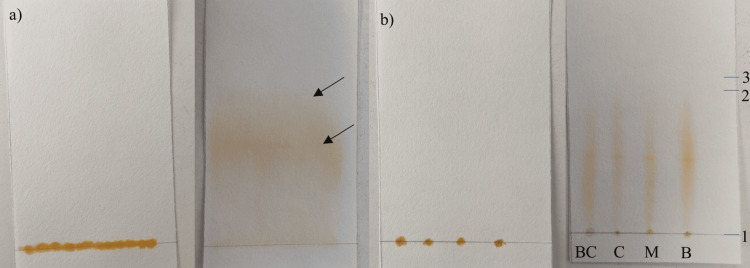
Paper chromatograms after the experiment a) Staphyloxanthin pigment is separated into two phases: a golden yellow phase (above) followed by an orange phase (below).  b) Analysis of extracted staphyloxanthin from various media (BC- Beetroot with carrot agar, C-Carrot agar, M- Milk agar, and B- Beetroot agar. 1- Origin spot, 2- Solute front, and 3- Solvent front. The retardation factor (Rf) of staphyloxanthin was found to be 0.95.

The paper chromatography analysis reveals the separation of staphyloxanthin pigment into two distinct bands (refer to Figure 12): a golden-yellow band positioned uppermost, followed by an orange band beneath it. The retardation factor (Rf) for the staphyloxanthin pigment across all compared media was determined to be 0.95, calculated using the formula: “Rf = Distance traveled by the solute front (4.3 cm)/Distance traveled by the solvent front (4.5 cm)” [15]. This Rf value was then compared with standard carotene values ranging from 0.91 to 0.99.

Analysis of gene expression

**Figure 13:**
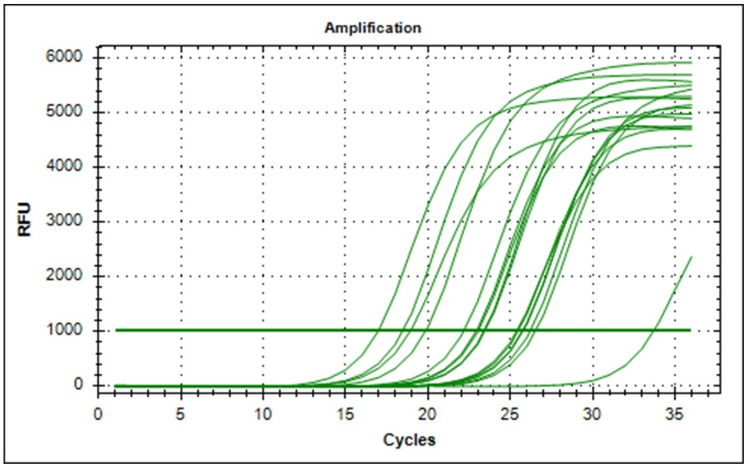
Representative graph showing crtM expression with reference gene (16srRNA) in S. aureus from various media

The presence of the staphyloxanthin synthesizing enzyme gene was confirmed through the utilization of the crtM gene primer. Investigation across various media types, including nutrient agar, milk agar, carrot agar, beetroot with carrot agar, and beetroot agar indicated consistent expression of the crtM gene (See Figure 13). Gene expression levels were evaluated for fold change using the 2^(-Delta-Delta CT) method by employing 16srRNA as internal control, which demonstrates a uniform crtM gene expression in *S. aureus* across the tested media.

## Discussion

Microbial pigments have gained significant attention in the industrial field, ranging from riboflavin, a food colorant, to antimicrobial products such as violacein. Bacterial pigments, in particular, have been recognized for their diverse pharmaceutical properties, including anti-inflammatory, anti-allergic, antimicrobial, and anti-cancer activities [16]. Various shades of bacterial pigments exist, such as red, orange, yellow, green, blue, and violet, but, golden yellow is uniquely associated with *S. aureus* staphyloxanthin.

Employing bacterial pigments instead of synthetic ones offers several advantages. These include their eco-friendly nature, wide range of activities, ease of propagation of bacteria in fermenters, availability of diverse strains, effortless manipulation of genes, sustainable production methods, and the utilization of inexpensive substrates for bacterial mass propagation [2]. Contrary to other bacterial pigments commonly employed in industries, staphyloxanthin pigment has received little attention in industrial microbiology due to its limited production in widely used media like nutrient agar. *S. aureus* is well known due to its severe infectious traits, but its possible applications were ignored in the industrial field of microbiology. Previous studies introduced milk agar to enhance staphyloxanthin production [10]. However, no alternative media is available for the enhancement of staphyloxanthin. Our study sheds light on the novel strategy of enhancing staphyloxanthin production.

**Figure 14:**
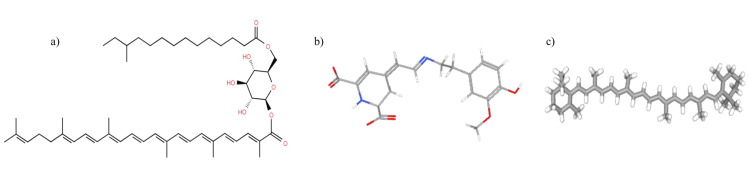
Carotenoids a) Structure of staphyloxanthin (Lipid Maps® Structure Database, LM ID-LMPR 0106010043). Due to its conjugated C=C, it interacts with free radicals and singlet oxygen generated by oxidative stress. b) Structure of betaxanthin (PubChem structure CID 135926572) in beetroot. c) Structure of beta-carotene (PubChem structure CID 5280489) in carrot.

Our observations revealed that basal media supplemented with beetroot and carrot powder effectively enhanced staphyloxanthin synthesis in *S. aureus*. The intensified pigment expression observed in colonies grown on beetroot and carrot agar can be attributed to betaxanthin and beta-carotene, respectively (refer to Figure 14). Notably, the shade of staphyloxanthin varied depending on the media utilized. To elucidate this variation, UV-visible spectroscopy was employed to analyze the spectrum shade. Results indicated a transition in the visible spectrum, shifting from 450 nm (yellow) on milk agar to 470 nm (yellow-orange) on carrot agar, and further to 480 nm (orange) on beetroot agar. However, the mechanism driving this color shift remains unknown.

Quantification of staphyloxanthin production via microtiter plate assay revealed that beetroot agar yielded the highest concentration, followed by milk agar and carrot agar. Surprisingly, the combination of beetroot and carrot agar resulted in an inefficient yield. To explore potential differences in the retardation factor, paper chromatography was utilized. Staphyloxanthin from various media exhibited a similar retardation factor. Additionally, the separation of staphyloxanthin into two bands resembled findings reported by Munera-Jaramillo et al., wherein an upper yellow band attributed to 4,4′-DNPA (4,4′-diaponeurosporenoic acid) and a lower orange band attributed to staphyloxanthin were observed [17].

Genotypic exploration of staphyloxanthin synthesis involved gene expression analysis of the enzyme crtM, which was found to be expressed in all the media used and the expression was similar across the various media used in this study. However, future research could extend this analysis to examine and compare the expression of other genes involved in staphyloxanthin synthesis, such as crtO, crtP, crtQ, and crtN, across different media. This study's limitation is the absence of practical demonstration of downstream processing and applications.

A deeper understanding of biosynthetic pathways, along with genetic modification and metabolic engineering, offers insights into utilizing recombinant DNA technology to enhance staphyloxanthin pigment production industrially. This natural golden pigment holds promise for applications in textiles and fabrics, providing resistance to fading and serving as a sustainable alternative to synthetic dyes. Moreover, its rich golden color, along with its UV-protective properties, makes it a suitable paint for use in walls and buildings. Taking advantage of its inherent antioxidant activity, future studies can explore incorporating staphyloxanthin into pharmaceutical products and cosmetics, thereby offering protection against oxidative stress induced by UV exposure and chemical damage [18].

Recently, the concept of green synthesis of nanoparticles utilizing microbial pigments has emerged, offering a wide range of applications such as anti-cancer and anti-bacterial agents, wastewater treatment, drug delivery, biodegradation, and biofuels [19]. In subsequent research, we can utilize staphyloxanthin for the synthesis of green nanoparticles and explore its potential for additional applications.

## Conclusions

Based on the findings of these experiments, it is evident that both beetroot agar and carrot agar have the potential to enhance pigment production and yield various shades in *S. aureus*. Subsequent studies could focus on scaling up the production of staphyloxanthin for its rich natural golden yellow-orange pigment, which holds promise for applications in the paint, textile, fabric, and food industries. Notably, due to its conjugated C=C chain structure, staphyloxanthin exhibits high antioxidant activity, effectively interacting with free radicals and reactive oxygen species generated by radiation exposure. This characteristic makes it a promising candidate for inclusion in sunscreen lotions and face creams, offering protection against radiation-induced skin damage. Additionally, these innovative cultural media could potentially be employed to enhance the production of carotenoids in other bacteria and fungi, including astaxanthin and zeaxanthin, for commercial applications. However, further research is warranted to validate these possibilities.
